# Chance or challenge, spoilt for choice? New recommendations on diagnostic and therapeutic considerations in hereditary transthyretin amyloidosis with polyneuropathy: the German/Austrian position and review of the literature

**DOI:** 10.1007/s00415-020-09962-6

**Published:** 2020-06-04

**Authors:** Maike F. Dohrn, Michaela Auer-Grumbach, Ralf Baron, Frank Birklein, Fabiola Escolano-Lozano, Christian Geber, Nicolai Grether, Tim Hagenacker, Ernst Hund, Juliane Sachau, Matthias Schilling, Jens Schmidt, Wilhelm Schulte-Mattler, Claudia Sommer, Markus Weiler, Gilbert Wunderlich, Katrin Hahn

**Affiliations:** 1 Neuromuscular Outpatient Clinic, Department of Neurology, Medical Faculty, RWTH Aachen University, Aachen, Germany; 2 Department of Orthopedics and Trauma Surgery, Medical University of Vienna, Vienna, Austria; 3 Division of Neurological Pain Research and Therapy, Department of Neurology, University Hospital Schleswig-Holstein, Campus Kiel, Kiel, Germany; 4 Department of Neurology, University Medical Center of the Johannes Gutenberg University, Mainz, Germany; 5 Department of Neurology, Red Cross Pain Centre Mainz, Mainz, Germany; 6 Department of Neurology, Faculty of Medicine and University Hospital Cologne, Cologne, Germany; 7 Department of Neurology, University Hospital Essen, Essen, Germany; 8 Amyloidosis Center Heidelberg, Heidelberg University Hospital, Heidelberg, Germany; 9 Department of Neurology, Heidelberg University Hospital, Heidelberg, Germany; 10 Department of Neurology with Institute of Translational Neurology, University Hospital of Muenster, Münster, Germany; 11 Department of Neurology, University Medical Center Göttingen, Göttingen, Germany; 12 Department of Psychiatry and Psychotherapy, University Hospital Regensburg, Regensburg, Germany; 13 Department of Neurology, University of Würzburg, Würzburg, Germany; 14 Center for Rare Diseases, Faculty of Medicine and University Hospital of Cologne, Cologne, Germany; 15 Department of Neurology, Charité University Medicine, Berlin, Germany

**Keywords:** TTR amyloidosis, Diagnostic intervals, Follow-up monitoring, Pre-symptomatic carriers, TTR stabilizers, Gene-silencing therapies

## Abstract

Hereditary transthyretin amyloidosis is caused by pathogenic variants (ATTR_v_) in the *TTR* gene. Alongside cardiac dysfunction, the disease typically manifests with a severely progressive sensorimotor and autonomic polyneuropathy. Three different drugs, tafamidis, patisiran, and inotersen, are approved in several countries, including the European Union and the United States of America. By stabilizing the TTR protein or degrading its mRNA, all types of treatment aim at preventing amyloid deposition and stopping the otherwise fatal course. Therefore, it is of utmost importance to recognize both onset and progression of neuropathy as early as possible. To establish recommendations for diagnostic and therapeutic procedures in the follow-up of both pre-symptomatic mutation carriers and patients with manifest ATTR_v_ amyloidosis with polyneuropathy, German and Austrian experts elaborated a harmonized position. This paper is further based on a systematic review of the literature. Potential challenges in the early recognition of disease onset and progression are the clinical heterogeneity and the subjectivity of sensory and autonomic symptoms. Progression cannot be defined by a single test or score alone but has to be evaluated considering various disease aspects and their dynamics over time. The first-line therapy should be chosen based on individual symptom constellations and contra-indications. If symptoms worsen, this should promptly implicate to consider optimizing treatment. Due to the rareness and variability of ATTR_v_ amyloidosis, the clinical course is most importantly directive in doubtful cases. Therefore, a systematic follow-up at an experienced center is crucial to identify progression and reassure patients and carriers.

**Electronic supplementary material:**

The online version of this article (10.1007/s00415-020-09962-6) contains supplementary material, which is available to authorized users.

## Introduction

Hereditary transthyretin amyloidosis was first described by the Portuguese neurologist Andrade in 1952 [1]. Based on the typical symptoms at onset, he named the disease “mal dos pézinhos” (painful feet). In 1978, Costa and colleagues identified abnormal prealbumin, which is identical with transthyretin (TTR), to be part of the amyloid deposits [2]. Both wild-type (ATTR_wt_) and mutant variants (ATTR_v_) of TTR amyloid can cause a systemic amyloidosis.

**Fig. 1:**
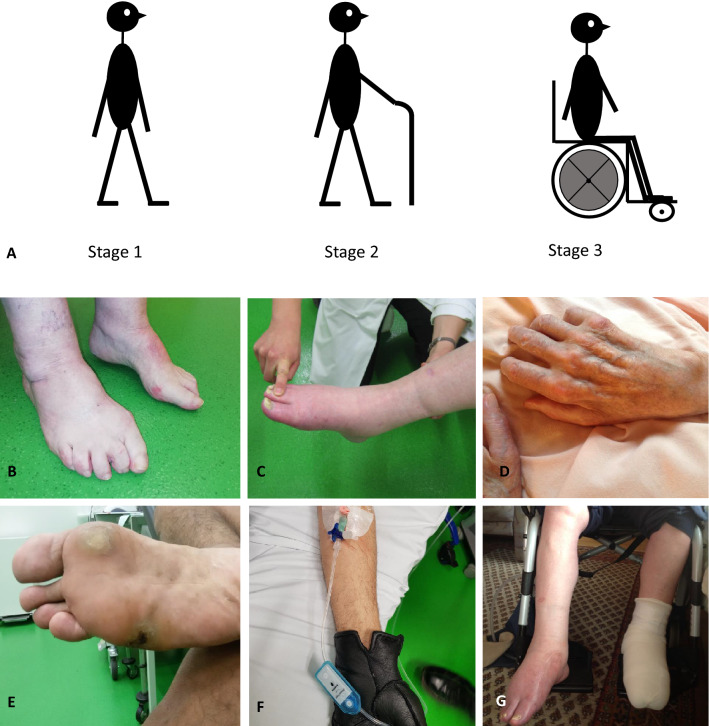
Coutinho disease stages. The natural course of ATTR_v_ amyloidosis-related polyneuropathy is traditionally divided into three stages. **a** A symptomatic patient, who is fully ambulant, is classified as stage 1. The need of one or two walking aids defines stage 2. Whenever a patient becomes wheelchair-bound or bedridden, an affected individual will be categorized as a stage 3 patient. The score does not depict cardiac and autonomic symptoms and cannot distinguish between a primarily motor gait disturbance and an afferent ataxia as the leading cause for ambulatory impairment. **b** A stage 1 patient with ankle edema and claw toes. **c** A stage 2 patient with weakness of toe elevation and ankle edema. **d** A bedridden stage 3 patient with advanced atrophies of the intrinsic hand muscles. **e** A stage 1 patient with non-healing wounds and previous toe amputations. **f** A stage 2 patient, who wears gloves to prevent painful skin contact. **g** The same patient as in C 2 years later: Wheelchair-bound, ankle edema, and non-healing wounds

ATTR_v_ amyloidosis is a rare, hereditary disease of autosomal dominant inheritance typically manifesting with a rapidly progressive sensorimotor and autonomic polyneuropathy, but also causing cardiac dysfunction, ocular, and gastrointestinal symptoms [3]. Relying on broad observatory studies of the Portuguese patient population, Coutinho and colleagues classified three disease stages (Fig. 1) based on walking capacity [4]: a symptomatic, but fully ambulatory patient is therefore in stage 1, the need for walking aids defines stage 2, and wheelchair dependence stage 3. To underline its systemic disease character, the international society of amyloidosis (ISA) replaced the previous disease name “familial amyloidotic polyneuropathy” (FAP) by the term “ATTR_v_ amyloidosis” in 2018 [5], in which “v” stands for variant and can be specified by the respective mutation. Depending on the leading manifestation, “with polyneuropathy” can optionally be added to refine this diagnosis.

More than 140 mutations in the *TTR* gene have been described to date, most of which are amyloidogenic [6]. By destabilizing the TTR tetramer, a circulating transport protein of thyroxine and retinol binding protein, some mutations foster its dissociation, the rate-limiting step of amyloidogenesis, while others facilitate misfolding and denaturation of monomers and therefore the irreversible formation of amyloid fibrils. Besides the already deposited amyloid fibrils, non-fibrillar circulating formations contribute to neurotoxicity and organ dysfunction [7, 8]. Comparing endemic and non-endemic areas, the clinical course varies distinctly even within carriers of the same *TTR* mutation [3, 9–11]. With an arbitrary cut-off at the age of 50 years, the typical early-onset and the mostly non-endemic late-onset type of disease are distinguished. The pathophysiological background of this phenotypic spectrum has not been fully understood to date. Depending on the regional (and individual) penetrance, some mutation carriers only develop first symptoms at the age of 70 years or older, whereas others remain asymptomatic within their entire life span. Indicators of an underlying ATTR_v_ amyloidosis are both a rapidly progressive sensory or sensorimotor neuropathy typically accompanied by autonomic dysfunction and cardiac and/or gastrointestinal symptoms [12, 13]. To confirm the diagnosis, a pathogenic mutation must be proven by *TTR* sequencing [14]. In Germany and Austria, amyloid deposits can, but do not necessarily have to be shown in tissue specimens, such as abdominal fat aspirates, salivary glands, myocardial biopsies, sural nerve, or skin specimens. Some patients are identified by occasional amyloid findings in tissue biopsies of other indication.

If untreated, the combination of heart failure, wasting, and secondary infections leads to death within 7–11 years after onset [15]. As more than 90% of circulating TTR is of hepatic origin, the first ever causative treatment option was liver transplant, which has successfully been conducted since 1990 [16, 17]. The replacement of mutant by wild-type TTR therefore constitutes the first form of genetic therapy, which significantly improved the patients’ life expectancy [17–20]. Based on the high risk of age- and stage-related complications, however, liver transplantation is not recommended in elderly patients and in those with an advanced disease stage or cardiac involvement. For younger patients as well, the procedures associated with the surgery and a life-long immunosuppression entail a high health burden. Besides, it is now known that the ATTR_v_-related symptoms can secondarily progress after liver transplant, which is due to seeding effects of previously deposited amyloid [21]. In a dosage of 20 mg per day, the TTR-stabilizing oral drug tafamidis meglumine has received its approval in the European Union (EU) for the treatment of stage 1 ATTR_v_ polyneuropathy in 2011. In several clinical trials, it showed to significantly slow down neurological and cardiological aspects of the disease course [22–24], which led to approval by the federal drug and food administration (FDA) for ATTR cardiomyopathy in a higher dosage of 60 mg in 2019. In 2018, two novel treatment approaches, the small interfering RNA patisiran [25] and the antisense oligonucleotide inotersen [26], were both approved in the EU and USA for the first and second disease stages of ATTR_v_ amyloidosis with polyneuropathy. Based on the highly specific degradation of *TTR* messenger RNA (mRNA) in hepatocytes, the serum TTR levels were significantly reduced by both drugs. Compared with placebo-treated patients, this resulted in a highly significant benefit concerning neuropathy progression and quality of life.

With the availability of causative treatment options (Table 1) that all aim at stopping or slowing down disease progression, it is of greatest importance to recognize ATTR_v_ amyloidosis as early as possible. Due to the rarity of the disease and frequent misdiagnoses in the first place, however, therapy can be fatally prolonged.

**Table 1:** Approved medications

	Tafamidis (Vyndaqel™)	Patisiran (Onpattro™)	Inotersen (Tegsedi™)
Countries with approval	European Union, Iceland, Norway, Liechtenstein, Japan, Argentina, Mexico, Israel, South Korea, Brazil, Hong Kong, Macau, Macedonia, Russia, Colombia, Serbia, United States of America, United Arab Emirates, Australia, Canada, Singapore, Switzerland	European Union, Switzerland, United States of America, Canada, Brazil, Japan	European Union, United States of America, Canada, Brazil
Approved in Europe since	2011	2018	2018
Approval limited to Coutinho stage	1, cardiomyopathy	1 and 2	1 and 2
Application	Oral	i.v.	s.c.
Frequency	1 ×/day	1 ×/3 weeks	1 ×/week
Pre-medication	None	Paracetamol, prednisolone, H1- and H2-receptor blockers	None
Endpoints of phase III trial	Stabilization of NIS-LL and quality of life	TTR knockdown by ~ 80%, improvement of mNIS+7, and quality of life	TTR knockdown by ~ 70%, stabilization of mNIS+7, and quality of life
Long-term data	~ 70% response rate	No data	No data
Significant effect on cardiomyopathy	Yes	Yes	Yes
Blood–brain barrier passage	Partial	None	None
Severe side effects	None	Infusion reactions	6 SAEs in the phase III trial: 3 × thrombopenia, 3 × glomerulonephritis

Synoptic summary on the three drugs tafamidis, patisiran, and inotersen all approved in Europe and the United States of America. The herein depicted data have been retrieved from the respective specialist information and all three phase III trials [22, 25, 26]

*i.v.* intravenous, *s.c.* subcutaneous, *H1/H2* histamine receptors, *NIS-LL* neuropathy impairment score for lower limbs, *mNIS+7* modified neuropathy impairment score with seven additional items, *SAE* serious adverse event

In this work, a group of German and Austrian neurologists experienced in the treatment of ATTR_v_ amyloidosis has joined their expertise and summarized the following comprehensive recommendations.

## Methodologies

The present work summarizes the current literature and the authors’ recommendations on diagnostic and therapeutic procedures in the neurological work-up of ATTR_v_ amyloidosis. Compiling the neurological consensus position of the German and Austrian interdisciplinary amyloidosis centers, it represents the non-endemic areas, in which ATTR_v_ is a very rare and often overseen disease. Located at 12 different centers, the authors currently treat approximately 250 patients in stage 1 or stage 2 with one of the approved drugs and further provide follow-up and care for about 100 stage 3 patients and 100 stage 0 mutation carriers. Additionally, they follow about 600 patients with ATTR_wt_ amyloidosis. Previous recommendations [27] have been implemented.

## Disease relevance

### Epidemiology

ATTR_v_ amyloidosis is considered a rare, an “orphan” disease. With known cases in more than 30 countries all over the globe [28], the local disease frequency is highly variable. “Endemic” areas with a particularly high prevalence are defined regions in northern Portugal and Sweden, where one in 1000–10,000 inhabitants would contract the disease [15].

In Germany and Austria, there are about 400 known disease cases to date [14]; however, several patients and families might be undiagnosed so far. The estimated prevalence varies from 1 to 5 cases in 1 million inhabitants [15, 28].

The most prevalent mutation worldwide is the exchange of the amino acid methionine for valine at protein position 50 (Val30Met following the traditional *TTR* classification; c.148G>A; p.Val50Met following the Human Gene Mutation Database), which accounts for about 99% of the Portuguese and 95% of the Swedish cases [15, 29]. In non-endemic countries, such as Germany, Austria, and Switzerland, ATTR_p.Val50Met_ amyloidosis causes about 40–60% of all cases relating to a greater genetic heterogeneity with more than 20 other known pathogenic variants [15]. Genotype–phenotype correlations provide a broad spectrum of disease manifestations partially correlating with the underlying variant. Some mutations (e.g., p.Val40Ile, p.Val142Ile) are preponderantly associated with a cardiomyopathy, whereas others (p.Val50Met) typically manifest with a leading neuropathy. The p.Leu75Pro variant is known to cause a particularly rapid and aggressive course [8, 30], while the two benign variants p.Arg124His and p.Thr139Met have a TTR-stabilizing effect even in compound heterozygosity with p.Val50Met, reducing fibril deposition and causing a milder clinical picture [31]. One genetic variant can, however, cause different onset phenotypes, and even within the same families, the symptom spectrum can be heterogeneous. Underlying genetic and external disease modifiers are not yet fully understood and merit further investigation.

Up to 25% of the elderly population (80 years and beyond) are estimated to have systemic amyloid deposits caused by wild-type TTR (ATTR_wt_) known for its amyloidogenicity even though not associated with mutations in the *TTR* gene [32]. In ATTR_wt_ amyloidosis, a probably still underdiagnosed disease in the elderly population, the clinical picture is typically dominated by a cardiomyopathy, the symptomatic impact of which ranges from asymptomatic to congestive heart failure. Men are more frequently affected than women. The presence and pattern of an associated polyneuropathy have so far not been systemically investigated in ATTR_wt_ amyloidosis. With its consequences for patient mobility, however, it merits further investigation and a cautious screening in clinical practice.

### Stages, scales, and scores

With recent upcoming treatment options, the classification of disease stages and the definition of progression have gained greater clinical relevance (Fig. 1). It is recommended that disease dynamics should be evaluated by trained and, if possible, always the same neurologists. A detailed overview on the relevant examinations and score validations for clinical practice is given in Table 3 and in the Supplementary Material.

Following Coutinho and colleagues [4], four stages (0–3) are commonly applied focusing on the patient’s ambulatory capacity with or without aids (Fig. 1). Accordingly, the Peripheral Neuropathy Disability (PND) score [33] consists of five stages, which sub-differentiate walking ability in more detail (e.g., one or two crutches required). Both scores focus on gait and only partially depict motor and sensory qualities, which can be somewhat unprecise in practice, for example, no difference is made between afferent ataxia or steppage as the leading cause of instability. Small fiber-associated sensory and autonomic symptoms are under-represented. In close correlation with the detailed neurological status, the Neuropathy Impairment Score (NIS) [34] comprises a range of 0–244 points depicting isolated muscle strength, deep tendon reflexes, and a distal qualitative sensory assessment. For a more extensive, but also more objective assessment of each patient’s status, the NIS score has further been developed now (mNIS+7), including quantitative sensory testing (QST), nerve conduction studies (NCS), and autonomic test results. In the phase III APOLLO [25] and NEURO-TTR [26] study protocols, the mNIS+7 score was used with slight modifications concerning test parameters and sum score values. Based on these protocols, it is therefore not possible to carry out head-to-head comparisons of the two trials or trial drugs.

In real-life practice beyond the clinical trials, it is important to know not only the defining marks but also the typical overall patterns of symptom development within the treatment-limiting Coutinho stages: asymptomatic mutation carriers, identified by predictive genetic testing, are classified as Coutinho stage 0. For these pre-symptomatic individuals, there is no treatment approved. The symptomatic stage 1 commonly manifests with painful dysesthesias, numbness, and/or a reduction of temperature and pain perception in feet, while the walking ability is still independent from aids. As thinly or unmyelinated small nerve fibers are most prone to early damage in ATTR_v_ amyloidosis, muscle strength and deep tendon reflexes are typically preserved in the very early stage 1; however, autonomic symptoms can already be present. With disease progression, sensory symptoms rise up to the lower legs and hands. Neuropathic pain is typically described as a burning sensation with worsening at night. The loss of proprioception and pallesthesia as well as progressive weakness initially affecting toe extensors, but rapidly involving lower legs and hands, indicate a large fiber involvement in the disease course. Disturbed wound healing can lead to severe painless ulcerations up to osteomyelitis and the need of amputations. Due to afferent ataxia, steppage, and orthostatic dysregulation, gait disturbances might require the need of walking aids. This is what defines the brink to stage 2. When a patient becomes wheelchair-bound or bedridden, stage 3 is reached meaning that all of the currently available treatment options are no longer approved.

Besides sensorimotor impairment, autonomic symptoms essentially contribute to the disease burden, which is, however, not stage defining. Especially in early-onset patients, symptoms such as early satiety, diarrhea, and unintended weight loss, incontinence, erectile dysfunction, disturbed sweating, and orthostatic intolerance can be the predominant or even first manifestations of the disease potentially causing life-threatening complications such as urogenital infections, cardiac arrhythmia, and wasting [27]. In terms of cardiac involvement, 80% of all ATTR_v_ patients develop an increased myocardial wall thickness leading to restrictive cardiomyopathy and cardiac conduction disturbances including arrhythmia. Severe ocular manifestations like vitreous opacities and trabecular obstruction affect about 5–10% of the patients in the first disease stage but become more prevalent over time [35, 36]. Renal impairment with proteinuria and/or a reduced glomerular filtration can additionally occur in the advanced disease course. When gastrointestinal mucosae are affected by amyloid deposition, this can contribute to disturbed resorption of nutrients additionally to the effects of autonomic neuropathy. In the sum, all of this can cause severe muscular atrophy, wasting, and an increased liability to infections [3, 10].

## Treatment options

ATTR_v_ amyloidosis is a progressive, disabling disease with a lethal course if untreated. This has not only been consistently confirmed by several studies on the natural disease course, but also by prospective data obtained from the placebo groups of several clinical trials [25, 26]. Optimizing both span and quality of life must therefore be the major goal for all diagnostic and therapeutic considerations on a causative and symptomatic level.

Treating a systemic disease further requires an overall interdisciplinary work-up in both diagnostic and therapeutic procedures. Expert boards including cardiologists, hematologists, gastroenterologists, ophthalmologists, genetic counselors, and neurologists, mostly located at the specialized centers, engage the assignment of optimizing individual treatment strategies.

Stopping disease progression is the key intention of causative treatment, including TTR stabilization and knockdown. In the presence of contraindications or if a disease stage has already been reached that is too advanced  for approved causative treatment options, this aim switches towards a best possible symptom control, including management of pain, ulcerations, infections, diarrhea, dyspnea, depression, and anxiety. Despite the sparse data availability [37], physiotherapy and rehabilitation are considered to be beneficial for patients suffering from ATTR_v_ amyloidosis with polyneuropathy as it has been shown for other hereditary neuropathies, such as Charcot–Marie–Tooth disease [38]. Physiotherapy is intended to maintain or improve strength and walking ability, to delay osteoarticular complications, and to preserve activities of daily living. Furthermore, an individually tailored orthosis treatment is mandatory. As part of a multidisciplinary rehabilitative approach, social medicine aspects should also be included.

An absolute requirement for the initiation of any causative treatment for stage 1 or 2 neuropathy is a pathogenic *TTR* mutation proven by genetic testing. For asymptomatic mutation carriers or patients, who are no longer ambulant, there is currently no approved treatment available. Other than in countries such as Portugal, the detection of amyloid in a tissue biopsy is not considered obligatory in Germany and Austria. A discontinuous distribution of amyloid deposits might lead to false-negative histopathological results, so that waiting for a positive biopsy result can even delay an early intervention. Performing biopsies is recommended, whenever the cause of neuropathy symptoms is unclear especially in the presence of differential diagnoses such as diabetes or alcohol consumption as well as “refractory” chronic inflammatory demyelinating polyneuropathy (CIDP), which is one of the most frequent misdiagnoses of ATTR_v_ amyloidosis [39, 40].

In the EU, USA, and other countries including Canada and Brazil, there are currently three drugs approved for the treatment of the ATTR_v_-related polyneuropathy (Table 1).

### TTR stabilization: tafamidis meglumine and diflunisal

The small molecule tafamidis meglumine (Vyndaqel™), an orally applicable benzoxazole, selectively binds human plasma TTR in the thyroxine binding groove, which has a stabilizing effect on the tetramer. By creating a kinetic barrier for tetramer dissociation, the rate-limiting step of amyloidogenesis, tafamidis intervenes in the early steps of pathophysiology [22, 41, 42]. In the EU, the approval for the 20 mg dosage became effective in 2011; however, it has so far been limited to the first Coutinho stage of ATTR_v_-related polyneuropathy meaning that patients need to be fully ambulatory without aids. In an 18-month lasting phase III trial, the intended stop of progression was realized in 60% of the treated compared to 38% of the placebo group [22]. Additionally, the modified body mass index (mBMI) and quality of life both showed a tendency towards stabilization under treatment, while continuously worsening in controls [22, 43]. An open-label extension trial with a time span of 5.5 years confirmed these data in a cohort of 71 patients [44]. The neurological outcome was measured by the neuropathy impairment score for lower limbs (NIS-LL), which turned out to increase significantly less in the verum compared with the placebo group. Accordingly, the nutritional status and quality of life were significantly better in the tafamidis group. Another trial showed a significant benefit in a smaller cohort of non-p.Val50Met patients as well [45]. The first long-term data on tafamidis in ATTR_v_-neuropathy patients revealed a negative correlation between the symptom severity at the beginning of treatment and the treatment response, pointing towards the necessity of an early diagnosis [23]. In October 2018, a new trial was published examining the 30-month effect of tafamidis in different dosages on the TTR-related cardiomyopathy including both hereditary and wild-type-associated forms of cardiac amyloidosis [24]. Primary outcome parameters were the overall survival and the reduction of cardiovascular events leading to hospitalization, secondary ones the clinical (6-min walk test) and functional (echocardiography, laboratory parameters) performance all being met from the 18-month visit on [24]. Tafamidis was well tolerated in all trials with a very low rate of side effects, such as vomiting or urinary tract infections. Since Mai 2019, tafamidis is approved in the USA for the treatment of both ATTR_v_- and ATTR_wt_-related cardiomyopathy, and in February 2020, the indication was accordingly broadened in Europe for the higher daily dosage of 60 mg.

Another medication with a stabilizing effect on the TTR tetramer is the non-steroidal antiphlogistic drug diflunisal, which structurally resembles tafamidis. A randomized, placebo-controlled trial including 130 patients with ATTR_v_ amyloidosis revealed in 2013 that diflunisal treatment can significantly reduce the NIS+7 score comparing the verum and placebo group over a time span of 2 years. A stabilization of neuropathy symptoms was reached in 29% of the verum and in 9% of the placebo group [46]. Due to its nephro- and cardiotoxic side effects and potential interactions with anticoagulant drugs, however, diflunisal has not been approved in Germany and Austria to date.

### Translational modification

By sequence-specific degradation of mRNA, gene-silencing therapies impede the translation of the intended target protein. This mechanism is independent from the underlying mutation inhibiting the overall hepatic production of the TTR protein. The drug has to be transferred to hepatocytes, where and where only it is meant to evolve its mRNA degrading effect. There are currently two gene-silencing drugs approved in the EU and USA for the treatment of polyneuropathy in ATTR_v_ amyloidosis, which differ in the way of reaching hepatocytes and the specific process of mRNA degradation, but both have a reduction of circulating TTR protein as their common effect mechanism.

#### Patisiran

The mechanism of patisiran (Onpattro™) is based on RNA interference (RNAi), which naturally happens in eukaryotic cells as part of the antiviral defense [47]. Patisiran consists of double-stranded oligonucleotides sized 21 base pairs in a lipid nanoparticle (LNP) formulation. It specifically recognizes and binds the complementary mRNA at the 3′ ending of the *TTR* gene, therefore inducing the so-called RNA-induced silencing complex (RISC). One of the major challenges is to deliver the substance to the target site, where it can then leap into action. To be selectively admitted to hepatocytes [48] and not to be destroyed by nucleases on the way, patisiran uses the aforementioned LNP layer, which fuses with endosomal membranes and therefore releases the oligonucleotides into cytoplasm.

In the APOLLO trial [25], an 18-month lasting, randomized, placebo-controlled phase III study with 225 participants, an intravenous application of 0.3 mg of patisiran per kg body weight every 3 weeks led to an 81% reduction (mean) of serum TTR. The primary endpoint, a stabilization of the mNIS+7 neuropathy score, was fully met. While placebo-treated patients worsened in a range of 28 ± 2.6 points, the verum cohort actually showed a clinical improvement with a point development of − 6 ± 1 points, which was statistically significant compared to both placebo and baseline. As most important secondary endpoint, the patients’ quality of life was accordingly improved [25]. Subgroup analyses additionally revealed an improvement of structural and functional cardiac markers (left ventricular hypertrophy, global longitudinal strain, N-terminal pro brain natriuretic peptide, and 10 m walk test) pointing out towards a positive effect of patisiran treatment on the ATTR_v_-related cardiomyopathy as well [49]. The most frequent side effects included peripheral edema and infusion reactions. In total, there were 13 deaths reported in the APOLLO trial, which were equally distributed in the placebo and the verum group and did not relate to the drug, but to the severity of the underlying disease itself.

In August 2018, patisiran received its approval in the EU and in the USA for the treatment of ATTR_v_-related polyneuropathy in Coutinho stages 1 and 2. The application is intravenous in a weight-dependent dosage of 0.3 mg/kg every 3 weeks. The infusion runs for about 80 min and requires a pre-medication with dexamethasone, paracetamol, and a combined H1/H2 receptor blockade. As the suppression of circulating TTR protein comes along with reduced vitamin A levels, a daily substitution is recommended in an oral dosage of 2500 IE. Home nursing programs have recently become available in some countries including Germany.

#### Inotersen

Inotersen (Tegsedi™) is a short, single-stranded, synthetic nucleic acid in a saline formulation, which selectively binds the *TTR* mRNA in the nucleus of hepatocytes and therefore activates the cell’s own RNases for degradation. Antisense oligonucleotides (ASOs) constitute a novel, but clinically yet established therapeutic concept for regulating protein expression and limiting toxic *gain*-*of*-*function* effects.

In the international phase III NEURO-TTR trial, 172 patients with stage 1 or 2 ATTR_v_-related polyneuropathy received inotersen or placebo in a 2:1 randomization. TTR serum levels were effectively reduced to about 25% (mean) of the baseline. Compared to placebo, the verum group showed a significantly lower mNIS+7 score and a significantly better quality of life in the Norfolk QoL score at week 66. These results were independent from the exact mutation, the Coutinho stage, and the presence or absence of cardiomyopathy [26]. Patients, who had previously been treated with tafamidis or diflunisal also showed a significant benefit from inotersen treatment. Focusing on the ATTR_v_-related cardiomyopathy, subgroup analyses showed a significant reduction of the left ventricular volume and septum diameter [50].

The most common side effects were nausea and vomiting, fever, glomerulonephritis, and alterations in the peripheral blood count. A relevant reduction of platelets occurred in about 60% of all inotersen-treated patients. One patient deceased due to intracranial hemorrhage associated with severe thrombopenia below 10/nl. It is therefore obligatory to monitor both blood cell counts and renal function on a regular basis (for details view expert information). It is further recommended to substitute vitamin A in a dosage of 2500 IE daily.

In July 2018, the EMA and FDA both approved inotersen for stage 1 and 2 ATTR_v_-related polyneuropathy. The application is subcutaneous in a weekly dosage of 284 mg. There is no pre-medication required.

### Choice of medication

Which of all available treatment options might be the best to begin with is an individual decision that has to be made on the basis of comorbidities and risk profiles. In stage 1 of neuropathy, all three medications come into question, while only patisiran and inotersen are approved for stage 2. It is not appropriate to maintain the first chosen drug until the brink of stage 2 comes to sight. To evaluate the need for and response to treatment, it is crucial to monitor the individual patient’s symptoms, to measure progression, and to recognize the earliest possible moment for switching therapeutic modalities [27].

In case of disease progression under therapy, it is necessary to consider changing the treatment modality as soon as possible. How exactly these switching algorithms look like, however, is a question that has so far not been addressed in the literature nor substantiated by trial data. If tafamidis is the first-line therapy of choice, either patisiran or inotersen should be chosen by individual risk constellations and patient’s preference. No scientific data support or refute the possibility that a patient progressive under one translation modification drug might respond to the other. It therefore remains an individual decision whether and how to switch in such case. Mechanistically, it appears not very likely that a patient progressive under RNA degradation treatment will respond to TTR stabilizers; however, the literature does not exclude this option neither. Halting one treatment due to side effects, might, however, be a different situation. If, for example, a stage 1 patient can no longer take patisiran or inotersen, this individual might still profit from tafamidis treatment.

## Diagnostic standards and follow-up monitoring

Standardized, interdisciplinary follow-up programs at experienced, networking centers are required to adequately address the heterogeneous penetrance and genotype–phenotype variations in terms of age at onset and organ involvement. These programs have to be sensitive enough on the one hand to assess the broad variety of disease manifestations, but on the other hand, they have to be dynamic and practicable for both patients and physicians. Local routines might, notwithstanding, be influenced by the endemic mutation spectrum [15, 51, 52]. Harmonizing the experience of the German and Austrian centers, it is recommended to monitor progression not focusing on one particular score only, but by repeatedly using a broad clinical approach, including a detailed patient history, clinical examinations, quantitative sensory testing (especially in the early disease phase), NCS (becomes representative within the course of stage 1), examinations of autonomic function (can be disturbed in the early course already), and specific questionnaires on autonomic disturbances, neuropathic pain, disability, and quality of life. A detailed overview on the recommended examinations is given in table 3 and in the supplementary material (Table 3).

Independent from the therapy of choice, it is always necessary to start as early as possible to prevent an irreversible nerve damage [53] and to achieve the best conceivable treatment response [25, 26, 44, 54]. *The point of symptom onset should therefore be the time of treatment onset as well.*

### Follow-up intervals

#### Asymptomatic mutation carriers

Due to the high variability in penetrance, a person with a positive predictive gene test result cannot be considered a “patient”, but an “individual at risk”. A clear distinction between pre-symptomatic “carriers” and symptomatic “patients” has therefore become a major diagnostic challenge. On the one hand, immediate recognition of the first signs of disease onset enables early intervention, which is essential for the patient’s prognosis. On the other hand, an important aim of follow-up procedures is to reassure individuals at risk typically having been closely involved in their relatives’ fatal disease course, by taking on the responsibility for symptom monitoring [55]. Depending on the underlying *TTR* mutation and the onset age in other affected family members, the predicted age at disease onset (PADO) might help estimate a carrier’s symptom onset as well [53]. It is recommended in the recent literature to start monitoring such an individual at risk about 10 years prior to PADO [53]. This can, however, be adapted to the intrafamilial variability and to the carrier’s personal demand.

As progression is one of the strongest red flag signs for disease onset in ATTR_v_ amyloidosis in order to distinguish from unspecific symptoms potentially related to other conditions or even to an increased introspection, it is helpful to dispose of long-term follow-up data gathered at one experienced center. It is herein recommended to schedule a detailed clinical follow-up at least once yearly (Table 2). A mutation carrier is considered symptomatic if subjectively reported complaints, including autonomic disturbances, sensory deficits, and/or neuropathic pain, come together with at least one objectifiable clinical or paraclinical test result, which is plausibly associated with ATTR_v_ amyloidosis [14]. Other “doubtful” constellations, such as asymptomatic carriers with carpal tunnel syndrome or potential neuropathy signs in NCS, merit tighter clinical monitoring. In case of differential diagnosis such as concomitant diabetes mellitus as well, a rapidly progressive disease course is the most relevant indicator of actual ATTR_v_ amyloidosis onset, which, again, requires regular follow-up examinations, for example, every three months.

**Table 2:** Recommended intervals for different types of visits and examinations

Coutinho stage	0	1	2	3	After change in medication
**Patient history** (recommended intervals in months)					
Sensorimotor symptoms	(6–) 12	6 (–12)	(3–) 6	(3–) 12	3
Autonomic symptoms	(6–) 12	6 (–12)	(3–) 6	(3–) 12	3
Medication	(6–) 12	6 (–12)	(3–) 6	(3–) 12	3
**Neurological examination** (recommended intervals in months)					
Qualitative sensory status	(6–) 12	6 (–12)	(3–) 6	(6–) 12	3
Distal muscle strength	(6–) 12	6 (–12)	(3–) 6	(6–) 12	3
Gait stability	(6–) 12	6 (–12)	(3–) 6	(6–) 12	3
Deep tendon reflexes	(6–) 12	6 (–12)	(3–) 6	(6–) 12	3
**Paraclinical examinations** (recommended intervals in months)					
Nerve conduction studies	12 (–24)	6 (–12)	6	–	6
Quantitative sensory testing	(6–) 12	6 (–12)	(6)	–	6
Skin conductance tests	(6–) 12	6 (–12)	(6)	–	6
Schellong’s test	(6–) 12	6 (–12)	6	–	3
**Clinical scores and questionnaires** (recommended intervals in months)					
NIS	12	6 (–12)	(3–) 6	(6–) 12	3
PND/Coutinho stages	12	6 (–12)	(3–) 6	(6–) 12	3
COMPASS-31	12	6 (–12)	(3–) 6	(6–) 12	3
R-ODS	–	6 (–12)	6	(6–) 12	6
Norfolk QoL	–	6 (–12)	6	(6–) 12	6

Summarized recommendations for examination intervals in the different disease stages. Pre-symptomatic carriers should at least be monitored once per year depending on the physician’s discretion and their own preference. Symptomatic patients are recommended to be seen every 6 months. With a growing disease burden, patients in stage 2 might need a tighter follow-up even, whereas with the loss of mobility, stage 3 patients might prefer not to come to the center more often than once per year. Independent from the disease stage, any shift in treatment modalities always requires a tight follow-up at least every 3 months until the disease progression is halted. Representing the first symptoms in an early stage 1, sensory and autonomic tests are of special interest, while nerve conduction studies can still be normal. With disease progression, however, quantitative sensory testing loses its specificity

#### Symptomatic patients

After beginning or switching treatment, it is recommended to monitor ATTR_v_ amyloidosis patients in three-month intervals. The focus of follow-up should be not only on the tolerability of the respective therapy, potential side effects, and the patient’s compliance, but also on the favored treatment response meaning a stabilization of symptom progression. Patients being stable under treatment should then undergo regular follow-up visits every 6 months in Coutinho stage 1 and every 3–6 months in Coutinho stage 2, at the treating neurologist’s discretion (Table 2). Any worsening of disease symptoms requires immediate changes in the diagnostic and therapeutic procedures: In cases of doubtful progression, it is recommended to increase the frequency of follow-up visits in order to better understand disease dynamics. If a patient is measurably progressive, however, a change in the therapeutic regimen has to be considered. It is neither necessary nor recommended to wait for a change in disease stage before adapting treatment, but to do so as soon as progression can be detected. As autonomic and cardiac symptoms are not fully depicted by the NIS score and its variants, by a change of which progression was defined in several clinical trials [22, 25, 26], the decision to switch the treatment modality does not necessarily require a pre-defined change in score points, but has to be evaluated based on the individual patient’s clinical course.

From a neurological point of view, the pedestal of every diagnostic work-up (Table 3) contains a detailed patient history, a clinical examination including the different modalities of the NIS score, questionnaires (e.g., R-ODS, COMPASS-31, and Norfolk Quality of Life), as well as NCS. If available, examinations, such as quantitative sensory testing (QST), Sudoscan, and sympathetic skin response (SSR), might contribute helpful additional information on small fiber impairment in the very early stage 1, when NCS are still insensitive. In advanced stages, a detailed clinical examination (NIS) might be sufficient if selectively added by NCS [56, 57]. As a systemic progression marker, the mBMI is easy to assess by multiplying the patient’s BMI with the current albumin level [g/l] in serum. By doing so, the nutritional status can be controlled and a potential bias caused by cardiac decompensation and peripheral edema is meanwhile addressed.

**Table 3:** Diagnostic steps in clinical trials and practice

Diagnostic method	Parameters of interest	Strengths	Weaknesses	Validation, use, and references
Patient history	Positive sensory symptoms: dysesthesia, paresthesia, neuropathic pain Negative sensory symptoms: numbness, thermal hypoesthesia, insensitivity to pain Distal or proximal muscle weakness Impaired fine motor skills Gait disturbances: stumbling, steppage, afferent ataxia, walking aids, falls Autonomic disturbances: orthostatic dysfunction, disturbed sweating, diarrhea and/or constipation, early satiety, unintended weight loss, incontinence, erectile dysfunction Cardiac symptoms: ankle edema, effort-dependent dyspnea, palpitations, dizziness Other organ involvement: carpal tunnel syndrome, vitreous opacities, or nephrotic syndrome Treatment side effects including specific (e.g. infusion reactions) and unspecific (e.g. headaches, urinary tract infections) reactions Family history	Easy, fast, non-invasive, and cheap to assess and to repeat Applicable in all disease stages Depicts a broad spectrum of disease aspects	Subjectivity	Recommendations on clinical practice [1–3]
Neurological examination	Sensory status: light touch, vibration, position, temperature, and pinprick sensation Gait patterns: afferent ataxia, steppage Isolated muscle strength (Medical Research Council) Deep tendon reflexes Inspection of the undressed extremities: wounds, ulcera, atrophy, edema	Easy, fast, non-invasive, and cheap to assess and to repeat Broad overview on different disease aspects Reliability and objectivity can be increased when performed by an experienced/the same examiner Applicable in all disease stages	Examiner dependency	NIS [4, 5] NIS-LL [6] mNIS+7 [5, 7, 8]
Nerve conduction studies	Neuropathy verification, analysis of patterns (length-dependent, sensorimotor) and qualities (axonal damage represented by reduced compound motor and sensory nerve action potentials) Pathological spontaneous activity in distal muscle electromyography as a sign of axonal damage Nerve conduction velocities may be delayed and F-waves abolished due to the loss of fast conduction axons, which can mislead to the diagnosis of CIDP [9]	Objective, quantifiable and repeatable tool to measure large fiber function Very sensitive in stage 1 and 2 Equipment is widely available	Uncomfortable/painful procedure Less sensitive in the very early stage 1 and from late stage 2 on	mNIS+7 [5, 7, 8]
Neuromuscular ultrasound	Cross-sectional areas are typically not or mildly enlarged only (other than in CIDP or demyelinating CMT) Increased muscle echogenicity in advanced disease stages	Objective, quantifiable tool to measure nerve structure Suitable to distinguish from CIDP Non-invasive, not painful	Requires specific equipment and training	Ultrasound pattern sum score (UPSS) [10]
Quantitative sensory testing	Early signs of small fiber involvement represented by cold detection threshold, mechanical pain threshold, paradoxical heat sensations (indicating dysfunction of Aδ fibers each), warm detection and heat pain threshold (indicating C fiber involvement), cold pain and pressure pain threshold (C and Aδ fibers) Distinction from (early) large fiber involvement (represented by mechanical detection and vibration threshold) and central sensitization (represented by dynamic mechanical allodynia and mechanical pain sensitivity)	Sensitive in very early stage 1 (small fiber involvement) Non-invasive, easy to learn	Less sensitive from late stage 1 on Highly dependent on the patient’s collaboration Time consuming Requires standardized equipment and training	DFNS protocol [11–14] mNIS+7 [5, 7, 8]
Autonomic testing	Orthostatic dysregulation (Winkler scale, Schellong test, tilt table) Disturbed sweating [SUDOSCAN, quantitative sudomotor axon reflexe testing (QSART)] Gastrointestinal disturbances (modified body mass index)	Sensitive already in the early disease stages	Relies partly on subjective patient descriptions Susceptible to interference, requires patient preparation (e.g. no caffeine, no skin care products) Tilt table: can cause uncomfortable effects like dizziness, fainting; not possible in cases of cardiac arrhythmia or pacemaker dependence	COMPASS-31 [7, 15] Winkler scale CASS score [16]
Questionnaires	Pain: neuropathic character, severity, course (PainDETECT, PainPREDICT, Neuropathic Pain Symptom Inventory (NPSI)) Disability: daily-life activities, impairment quantification (Rasch-built Overall Disability Scale (R-ODS)) Quality of life (Norfolk QoL)	Easy to assess, non-invasive, repeatable	Subjectivity	PainDETECT [17] NPSI [18] PainPREDICT [19] R-ODS [7, 8, 20, 21] Norfolk QoL [7, 8, 22, 23]
Histology	Congo red amyloid staining: amyloid depiction, most frequently used; not obligatory for treatment, helpful in undecisive cases Thioflavine S fluorescence microscopy: amyloid depiction ATTR immunohistochemistry: TTR specification	Salivary glands [24] and fat tissue aspirates [25] are easy accessible and show a high sensitivity Skin biopsies provide additional information on small fiber degeneration Sural nerve (and muscle) biopsies are helpful to distinguish from other neuropathies Myocardial biopsies might be obtained in the standard diagnostic procedures of cardiomyopathy workup Other potentially assessable tissues: deep rectal mucosa, carpal ligament material	Risk of false negative test results Should be evaluated in specified centers only	Amyloidosis guidelines and phenotyping studies [26, 27]

Overview on the different diagnostic work steps, including parameters of interest, validation, strengths, and weaknesses. A detailed description of all diagnostic procedures and a list of the cited references are provided in the supplementary material

Based on individual needs, the exchange of information and counseling plays an important role in the diagnostic and follow-up procedures in patients with ATTR_v_ amyloidosis and relatives both being potential caregivers and individuals at risk. It is somewhat challenging and requires extra time to educate, but not to frighten both patients and carriers. An inadequate emotional repression or lack of knowledge might delay the recognition of disease onset, while an exceeding self-observation triggers psychosomatic symptoms potentially disguising ATTR_v_ amyloidosis-related complaints as well. Particularly in larger kindreds with long pedigrees of yet deceased antecedents, it is helpful to involve a human geneticist or psychologist experienced in the counseling of fatal diseases.

It is recommended to regularly examine, treat, and counsel all carriers, patients, and caregivers at a local specialized center. These centers are due to forming networks on a national and international basis enabling the low-threshold exchange of knowledge and the participation in clinical trials.

## Summary

Hereditary transthyretin (ATTR_v_) amyloidosis is a rare, autosomal dominant disease with a progressively disabling and fatal course if untreated. Besides the invasive liver transplantation, three highly specific causative treatment options based on either protein stabilization or mRNA degradation have been approved so far.

In this work, a group of German and Austrian neurologists elaborated their harmonized recommendations for the diagnostic and therapeutic work-up for both pre-symptomatic mutation carriers and symptomatic patients with ATTR_v_ amyloidosis-related polyneuropathy.

The overall aim of any causative and symptomatic treatment is the best possible control of disease symptoms. Therefore, it is crucial to start a medication as early as possible. This requires an early recognition of disease onset and a tight monitoring of treatment response. The first-line medication should be chosen individually depending on the respective patient’s needs and contra-indications. In case of disease progression under therapy, the treatment modality should be optimized with the least possible delay. Progression, however, cannot be defined by one score or classification but has to be determined by experienced physicians considering both patient-reported symptoms, clinical signs, and measurable test results. Being a systemic disease, however, ATTR_v_ amyloidosis merits an interdisciplinary approach with regular follow-up visits at an experienced center.

## Electronic supplementary material

Below is the link to the electronic supplementary material.

Supplementary material: Detailed description of all recommended diagnostic steps and reference list for Table 3. (PDF 195 kb)
