# Supplementary material for: Chance or challenge, spoilt for choice? New recommendations on diagnostic and therapeutic considerations in hereditary transthyretin amyloidosis with polyneuropathy: the German/Austrian position and review of the literature
Source: J Neurol. 2020 Jun 4;268(10):3610–25. doi: 10.1007/s00415-020-09962-6 (PMC8463516; doi:10.1007/s00415-020-09962-6)
Supplement: Supplementary file 1 — Supplementary material: Detailed description of all recommended diagnostic steps and reference list for Table 3. (PDF 195 kb) [file 415_2020_9962_MOESM1_ESM.pdf]

Supplementary material

Chance or challenge, spoilt for choice? New recommendations on diagnostic and therapeutic considerations in hereditary transthyretin amyloidosis with polyneuropathy – the German/Austrian position and review of the literature

Maike F. Dohrn[1], Michaela Auer-Grumbach[2], Ralf Baron[3], Frank Birklein[4], Fabiola Escolano-Lozano[4], Christian Geber[5], Nicolai Grether[6], Tim Hagenacker[7], Ernst Hund[8, 9], Juliane Sachau[3], Matthias Schilling[10], Jens Schmidt[11], Wilhelm Schulte-Mattler[12], Claudia Sommer[13], Markus Weiler [8,9], Gilbert Wunderlich[6, 14], Katrin Hahn[15]

[1] Department of Neurology, Medical Faculty, RWTH Aachen University, Aachen, Germany

[2] Department of Orthopedics and Trauma Surgery, Medical University of Vienna, Vienna, Austria

[3] Division of Neurological Pain Research and Therapy, Department of Neurology, University Hospital Schleswig-Holstein, Campus Kiel, Kiel, Germany

[4] Department of Neurology, University Medical Center of the Johannes Gutenberg University, Mainz, Germany

[5] Red Cross Pain Centre Mainz, Department of Neurology, Mainz, Germany

[6] Department of Neurology, Faculty of Medicine and University Hospital Cologne, Cologne, Germany

[7] Department of Neurology, University Hospital Essen, 45147, Essen, Germany

[8] Amyloidosis Center Heidelberg, Heidelberg University Hospital, Heidelberg, Germany

[9] Department of Neurology, Heidelberg University Hospital, Heidelberg, Germany

[10] Department of Neurology with Institute of Translational Neurology, University Hospital of Muenster, Muenster, Germany

[11] Department of Neurology, University Medical Center Göttingen, Göttingen, Germany

[12] Department of Psychiatry and Psychotherapy, University Hospital Regensburg, Regensburg, Germany

[13] Department of Neurology, University of Würzburg, Würzburg, Germany

[14] Center for Rare Diseases, Faculty of Medicine and University Hospital of Cologne, Germany

[15] Department of Neurology, Charité University Medicine, Berlin, Germany

Detailed description of recommended diagnostic tests

Patient history

A detailed patient history is the core directive for every other type of diagnostic test [1-3]. By assessing a detailed patient history, one can already retrieve some hypotheses concerning the presence or absence of sensory, motor, and autonomic neuropathy symptoms and therefore enable the recognition of disease onset or progression. Positive sensory symptoms include dysesthesia, paresthesia, and neuropathic pain, examples for negative sensory symptoms are numbness, thermal hypoesthesia, and insensitivity to pain. The documentation should further include the presence of distal or proximal muscle weakness, impaired fine motor skills, and gait disturbances such as stumbling, steppage, afferent ataxia, walking aids, and

falls. Autonomic symptoms should be addressed as well by asking for orthostatic dysfunction, disturbed sweating, diarrhea and/or constipation, early satiety, unintended weight loss, incontinence, or erectile dysfunction. The presence of ankle edema, effort-dependent dyspnea, palpitations, or dizziness might point towards a cardiac involvement that merits further evaluation by a cardiologist. Other organ involvement such as carpal tunnel syndrome, vitreous opacities, or nephrotic syndrome can also be disclosed by a systematic assessment of symptoms. If a patient receives any causative or symptomatic treatment, the treating physician should always ask for specific (e.g. infusion reactions) and unspecific (e.g. headaches, urinary tract infections) side effects. It is furthermore important to query the family history, the patient's professional status as well as aspects of domestic care and the need for symptomatic medications.

Neurological examination

As an easy, fast, cheap, and non-invasive method, the physical examination still constitutes the most important approach to evaluate clinical symptoms and to recognize progression. Being the core part of different clinical scores (NIS [4,5], NIS-LL [6], mNIS+7 [5,7,8]), it has therefore become irreplaceable for the definition of clinical outcome parameters in clinical trials. In patients with $ATTR_v$ amyloidosis, the neurological examination is indicated in presumably asymptomatic mutation carriers as well as in all symptomatic disease stages. It comprises a detailed assessment of the sensory status differentiating the modalities of light touch, vibration, position, temperature, and pinprick sensation, a documentation of gait patterns including afferent ataxia and steppage, an examination of isolated muscle strength and of deep tendon reflexes. Additionally, it includes an inspection of the undressed extremities searching for non-healing wounds or ulcerations, for atrophies, or edema. Slight signs of progression are easier to evaluate if the examiner is experienced and, if possible, not changing between visits. Particularly in phases of subjective, but maybe not yet measurable disease progression, we recommend to repeat a detailed clinical examination at least every three months. In more stable disease stages, the intervals may be enlarged onto six to 12 months. When a patient becomes wheelchair- or bedridden, however, some parts of the clinical assessment will no longer be possible.

Nerve conduction studies (NCS)

NCS address the questions whether there is a measurable large fiber polyneuropathy, whether it preponderantly affects sensory or motor nerves, whether it follows a certain pattern of distribution, and whether it is of leading axonal or demyelinating quality. If performed repeatedly, it gives information on the neuropathy's progressiveness. In stage 1 $ATTR_v$ amyloidosis, NCS constitute an integral part of diagnostic routines both at the time of first diagnosis and in the follow-up and evaluation of treatment response. Per definition, there are no signs of large fiber damage in stage 0; and also in the very first phase of stage 1, there is typically a pure small fiber neuropathy. However, in the clinical course, a progressive involvement of large sensory nerves and later of motor nerve fibers becomes more and more evident. The typical $ATTR_v$ amyloidosis-associated polyneuropathy is length-dependent and of axonal

quality. By detection of pathological spontaneous activity first of all in the most distal muscles, the electromyography provides additional information on an acute axonal damage as part of the initial and early follow-up diagnostics. Low conduction velocities or delayed F-waves have been described in some cases of $ATTR_v$ amyloidosis, which can mislead to the diagnosis of chronic inflammatory demyelinating polyneuropathy (CIDP) [9]. As an additional tool, the nerve ultrasound may serve as a marker to distinguish CIDP and $ATTR_v$ amyloidosis by comparing the nerves' cross-sectional areas [10].

Especially in the first Coutinho stage, NCS constitute an important functional marker of progression. It is therefore recommended to repeat these examinations in intervals of six or, in clinically stable patients, of 12 months. In an advanced disease stage, the axonal integrity has typically been lost to such an extent that NCS are technically no longer informative.

Prior to the onset of an $ATTR_v$ amyloidosis-related polyneuropathy, a mostly bilateral carpal tunnel syndrome can commonly be demonstrated by NCS. It constitutes a soft tissue manifestation, which is so far beyond the label of all approved treatment approaches.

Quantitative sensory testing

The German Research Network on Neuropathic Pain (Deutscher Forschungsverbund Neuropathischer Schmerz, DFNS) has established a standardized protocol for quantitative sensory testing (QST) using calibrated cutaneous stimuli to delineate the type of nerve fiber involvement and the degree of degeneration compared to age-, sex- and body-site-dependent reference values [14,11,13,12]. By following this protocol, one can assess thermal detection and pain thresholds, paradoxical heat sensations, mechanical detection thresholds to von Frey filaments, mechanical pain thresholds to pinprick stimuli and blunt pressure, stimulus/response-functions for pinprick and dynamic mechanical allodynia, pain summation (wind-up ratio) and vibration detection thresholds. The combination of these values enables a functional evaluation of small and large nerve fibers [13], which makes it especially valuable in the very early stage 1 of $ATTR_v$ amyloidosis-related neuropathy, when NCS have not yet become informative.

Autonomic testing

Besides a sensorimotor neuropathy, autonomic symptoms are typical and can even be leading as first manifestation or main reason of disability in $ATTR_v$ amyloidosis [28]. The COMPASS-31 questionnaire provides an autonomic symptom score from 0 to 100 including 31 items within 6 domains [15]. As an alternative score, the compound autonomic dysfunction test (CADT) has been particularly developed for $ATTR_v$ amyloidosis [29]. Additionally, the Winkler scale queries autonomic symptoms using ten numeric and two additional nominal items to raise the suspicion of an orthostatic intolerance.

An easy bedside test to assess an orthostatic dysfunction is the Schellong test that compares heart rate and blood pressure in supine and upright position. If indecisive, the tilt table test is helpful to minimize

the effect of the muscle pump: By elevating the upper body, a sympathetic response is physiologically expected to occur compensating the increased hydrostatic pressure and therefore reduced centrally available blood volume. In case of an autonomic hypotension, a too low or excessive heart rate or significant decrease in blood pressure (> 20 mmHg systolic, > 10 mm Hg diastolic) refers to a pathological response. Moreover, cardiovagal indices (heart-rate variability at rest, forced breathing, Valsalva manoeuvre, and orthostasis) are measured to assess parasympathetic functioning. Within three to four minutes, the SUDOSCAN™ measures electrochemical skin conductance (ESC) by reverse iontophoresis and therefore provides information on a sudomotor dysfunction at palms and soles. The quantitative sudomotor axon reflex testing (QSART) measures the localized sweat production transmitted by axon reflexes crosslinking adjacent skin areas, which have previously been stimulated by a quantified electric impulse. Summarizing the results of the tilt table and QSART, the Composite Autonomic Severity Score (CASS) can be used to estimate the presence and severity of sympathoneural and cardiovagal dysfunction [16]. The autonomic function is considered normal with 0 points, mildly disturbed with one to three points, moderately disturbed with four to six points, and severely affected with seven to ten points.

Questionnaires

Especially the early symptoms of the $ATTR_v$-related polyneuropathy are typically very subjective, and measuring them and their individual progression appears somewhat challenging. It is therefore recommended to substantiate the patient history by adding standardized and validated questionnaires to each clinical visit. We will shortly propose some questionnaires on the following topics:

- Pain: Out of numerous pain scales, the painDETECT successfully asserted itself in clinical practice [17]. It contains 38 points in three sections: section 1 addressing pain intensity, character, and triggers, section two focusing on pain dynamics, and section three on its radiation. With an overall score of 19 points or more, neuropathic pain is likely with a probability of 90%, whilst it is unlikely when less than 12 points are summarized. With a sensitivity and specificity of 84% each, the painDETECT is a fast and easy tool to identify neuropathic pain at the brink between stage 0 and 1. It has, however, not been validated to measure pain progression, which should rather be addressed with the painPREDICT questionnaire or Neuropathic Pain Symptom Inventory (NPSI) for instance.

- Disability: The Rasch-built Overall Disability Scale (R-ODS) assesses the current physical function and disability by systematically inquiring 24 daily-life activities such as dressing, bathing, and grocery shopping. On a scale from 0 to 2, the patient is asked to graduate to what extent these activities can be executed. With an overall score of 0 points, the patient reaches the lowest, with 48 points the highest level of self-sufficiency. This score has previously been used in the context of different other hereditary and acquired neuropathies [20,21] and also took part in the phase III trials on patisiran and inotersen [7,8].

o Quality of life: The Norfolk Quality of Life (Norfolk QoL) questionnaire was originally established for diabetic neuropathies [22], but has been validated for the $ATTR_v$ amyloidosis-related polyneuropathy as well [23]. Comprising 35 items in total, the five domains depict the function of large and small nerve fibers, autonomic symptoms, and daily-life activities. The Norfolk QoL was part of the phase III clinical trials of tafamidis, patisiran, and inotersen, respectively.

Histological amyloid depiction

In the German speaking countries, it is not obligatory to histologically prove the presence of amyloid deposits before starting any treatment, if a patient is in a clear stage 1 or 2 of $ATTR_v$ amyloidosis-related polyneuropathy, which already includes a positive genetic test result. Tissue biopsies are helpful [27,26], however, in unclear cases with no previously affected relatives or if confounding comorbidities such as diabetes mellitus might as well be held responsible for neuropathy symptoms. Specimens from salivary glands (sensitivity 91% [24]) and abdominal fat aspirates (sensitivity 80-93% [25]) can easily and repeatedly be obtained. Besides Congo red staining for amyloid depiction, skin biopsies provide additional information on the intraepidermal nerve fiber density, whereas the more invasive sural nerve biopsies show the quality and extent of distal sensory nerve involvement. In the diagnostic workup of cardiomyopathy, a myocardial biopsy might be decisive to distinguish between light chain and wildtype TTR amyloidosis. Other tissues suitable for histological examinations are deep rectal mucosa as well as carpal ligament material.

References cited in table 3 and in the supplementary material:

1. Hund E, Kristen AV, Auer-Grumbach M, Geber C, Birklein F, Schulte-Mattler W, Sommer C, Schmidt H, Röcken C (2018) Transthyretin-Amyloidose (ATTR-Amyloidose): Empfehlungen zum Management in Deutschland und Österreich. Aktuelle Neurologie 45 (08):605-616

2. Adams D, Ando Y, Beirão JM, Coelho T, Gertz MA, Gillmore JD, Hawkins PN, Lousada I, Suhr OB, Merlini G (2020) Expert consensus recommendations to improve diagnosis of ATTR amyloidosis with polyneuropathy. J Neurol (doi: 10.1007/s00415-019-09688-0):1-14

3. Obici L, Kuks JB, Buades J, Adams D, Suhr OB, Coelho T, Kyriakides T (2016) Recommendations for presymptomatic genetic testing and management of individuals at risk for hereditary transthyretin amyloidosis. Curr Opin Neurol 29 (Suppl 1):S27

4. Dyck PJ, Sherman WR, Hallcher LM, John Service F, O'Brien PC, Grina LA, Palumbo PJ, Swanson CJ (1980) Human diabetic endoneurial sorbitol, fructose, and myo-inositol related to sural nerve morphometry. Ann Neurol 8 (6):590-596

5. Dyck PJB, González-Duarte A, Obici L, Polydefkis M, Wiesman J, Antonino I, Litchy WJ, Dyck PJ (2019) Development of measures of polyneuropathy impairment in hATTR amyloidosis: From NIS to mNIS+ 7. J Neurol Sci:116424

6. Coelho T, Maia LF, da Silva AM, Cruz MW, Planté-Bordeneuve V, Lozeron P, Suhr OB, Campistol JM, Conceição IM, Schmidt HH-JJN (2012) Tafamidis for transthyretin familial amyloid polyneuropathy: a randomized, controlled trial. Neurology 79 (8):785-792

7. Adams D, Gonzalez-Duarte A, O'Riordan WD, Yang C-C, Ueda M, Kristen AV, Tournev I, Schmidt HH, Coelho T, Berk JL (2018) Patisiran, an RNAi therapeutic, for hereditary transthyretin amyloidosis. N Engl J Med 379(1) (1):11-21

8. Benson MD, Waddington-Cruz M, Berk JL, Polydefkis M, Dyck PJ, Wang AK, Planté-Bordeneuve V, Barroso FA, Merlini G, Obici L (2018) Inotersen treatment for patients with hereditary transthyretin amyloidosis. N Engl J Med 379 (1):22-31

9. Lozeron P, Mariani L-L, Dodet P, Beaudonnet G, Théaudin M, Adam C, Arnulf B, Adams D (2018) Transthyretin amyloid polyneuropathies mimicking a demyelinating polyneuropathy. Neurology 91 (2):e143-e152

10. Grimm A, Axer H, Heiling B, Winter N (2018) Nerve ultrasound normal values–readjustment of the ultrasound pattern sum score UPSS. Clin Neurophysiol 129 (7):1403-1409

11. Geber C, Klein T, Azad S, Birklein F, Gierthmühlen J, Huge V, Lauchart M, Nitzsche D, Stengel M, Valet M (2011) Test–retest and interobserver reliability of quantitative sensory testing according to the protocol of the German Research Network on Neuropathic Pain (DFNS): a multi-centre study. Pain 152 (3):548-556

12. Magerl W, Krumova EK, Baron R, Tölle T, Treede R-D, Maier C (2010) Reference data for quantitative sensory testing (QST): refined stratification for age and a novel method for statistical comparison of group data. Pain 151 (3):598-605

13. Maier C, Baron R, Tölle T, Binder A, Birbaumer N, Birklein F, Gierthmühlen J, Flor H, Geber C, Huge V (2010) Quantitative sensory testing in the German Research Network on Neuropathic Pain (DFNS): somatosensory abnormalities in 1236 patients with different neuropathic pain syndromes. Pain 150 (3):439-450

14. Rolke R, Baron R, Maier Ca, Tölle T, Treede R-D, Beyer A, Binder A, Birbaumer N, Birklein F, Bötefür I (2006) Quantitative sensory testing in the German Research Network on Neuropathic Pain (DFNS): standardized protocol and reference values. Pain 123 (3):231-243

15. Sletten DM, Suarez GA, Low PA, Mandrekar J, Singer W COMPASS 31: a refined and abbreviated Composite Autonomic Symptom Score. In: Mayo Clin Proc, 2012. vol 12. Elsevier, pp 1196-1201

16. Sletten DM, Weigand SD, Low PA (2010) Relationship of Q-sweat to quantitative sudomotor axon reflex test (QSART) volumes. Muscle nerve 41 (2):240-246

17. Freynhagen R, Tölle TR, Gockel U, Baron R (2016) The painDETECT project–far more than a screening tool on neuropathic pain. Curr Med Res Opin 32 (6):1033-1057

18. Bouhassira D, Attal N, Fermanian J, Alchaar H, Gautron M, Masquelier E, Rostaing S, Lanteri-Minet M, Collin E, Grisart J (2004) Development and validation of the Neuropathic Pain Symptom Inventory. Pain 108 (3):248

19. Tölle T, Baron R, de Bock E, Junor R, Dias BC, Marshall S, Arnould B, Freynhagen R (2019) painPREDICT: first interim data from the development of a new patient-reported pain questionnaire to predict treatment response using sensory symptom profiles. Curr Med Res Opin 35 (7):1177

20. Roevekamp F, van Paassen B, Shy M, Reilly M, Kirk C, Bacon C, Davidson E, Linssen W, Faber C, Merkies I Rasch-built overall disability scale (R-ODS) for hereditary motor and sensory neuropathy (RODS-CMT). In: Journal of the Peripheral Nervous System, 2015. vol 2. WILEY-BLACKWELL, pp 221-222

21. Quan D, Adams D, Gonzalez-Duarte A, Polydefkis M, Kristen A, Tournev I, Schmidt H, Coelho T, Berk J, Gandhi P Evaluating the impact of Patisiran on disability using the Rasch-built overall disability scale (R-ODS) in patients with hereditary transthyretin-mediated (hATTR) amyloidosis in the APOLLO study. In: Muscle nerve, 2018. WILEY 111 RIVER ST, HOBOKEN 07030-5774, NJ USA, pp S51-S51

22. Vinik EJ, Hayes RP, Oglesby A, Bastyr E, Barlow P, Ford-Molvik SL, Vinik AI (2005) The development and validation of the Norfolk QOL-DN, a new measure of patients' perception of the effects of diabetes and diabetic neuropathy. Diabetes Technol Ther 7 (3):497-508

23. Vinik EJ, Vinik AI, Paulson JF, Merkies IS, Packman J, Grogan DR, Coelho T (2014) Norfolk QOL-DN: validation of a patient reported outcome measure in transthyretin familial amyloid polyneuropathy. J Peripher Nerv Syst 19 (2):104-114

24. Do Amaral B, Coelho T, Sousa A, Guimarães A (2009) Usefulness of labial salivary gland biopsy in familial amyloid polyneuropathy Portuguese type. Amyloid 16 (4):232

25. Gameren IIV, Hazenberg BP, Bijzet J, Rijswijk MHV (2006) Diagnostic accuracy of subcutaneous abdominal fat tissue aspiration for detecting systemic amyloidosis and its utility in clinical practice. Arthritis Rheum 54 (6):2015-2021

26. Dohrn MF, Röcken C, De Bleecker JL, Martin J-J, Vorgerd M, Van den Bergh PY, Ferbert A, Hinderhofer K, Schröder JM, Weis JJJon (2013) Diagnostic hallmarks and pitfalls in late-onset progressive transthyretin-related amyloid-neuropathy. J Neurol 260 (12):3093-3108

27. Röcken C, Ernst J, Hund E, Michels H, Perz J, Saeger W, Sezer O, Spuler S, Willig F, Schmidt H (2006) Interdisciplinary guidelines on diagnosis and treatment for extracerebral amyloidoses--published by the German Society of Amyloid Diseases (www. amyloid. de). Deutsche Medizinische Wochenschrift 131 (27 Suppl 2):S45-66

28. Kim DH, Zeldenrust SR, Low PA, Dyck PJ (2009) Quantitative sensation and autonomic test abnormalities in transthyretin amyloidosis polyneuropathy. Muscle nerve 40 (3):363-370

29. Denier C, Ducot B, Husson H, Lozeron P, Adams D, Meyer L, Said G, Plante-Bordeneuve V (2007) A brief compound test for assessment of autonomic and sensory-motor dysfunction in familial amyloid polyneuropathy. J Neurol 254 (12):1684-1688
